# Effect of oral administration of ethanolic extract of *Vitex negundo* on thioacetamide-induced nephrotoxicity in rats

**DOI:** 10.1186/1472-6882-13-294

**Published:** 2013-10-30

**Authors:** Farkaad A Kadir, Normadiah M Kassim, Mahmood A Abdulla, Wageeh A Yehye

**Affiliations:** 1 Department of Anatomy, Faculty of Medicine, University of Malaya, Kuala Lumpur 50603, Malaysia; 2 Department of Biomedical Science, Faculty of Medicine, University of Malaya, Kuala Lumpur 50603, Malaysia; 3 Nanotechnology & Catalysis Research Centre, (NANOCAT), University of Malaya, Block 3A, Institute of Postgraduate Studies Building, Kuala Lumpur 50603, Malaysia

**Keywords:** *Vitex negundo*, Thioacetamide, Urea, Creatinine, Histopathology, Immunohistochemistry, Nephrotoxicity

## Abstract

**Background:**

Oxidative stress due to abnormal induction of reactive oxygen species (ROS) molecules is believed to be involved in the etiology of many diseases. Evidences suggest that ROS is involved in nephrotoxicity through frequent exposure to industrial toxic agents such as thioacetamide (TAA). The current investigation was designed to explore the possible protective effects of the leaves of *Vitex negundo*(VN) extract against TAA-induced nephrotoxicity in rats.

**Methods:**

Twenty four *Sprague Dawley*rats were divided into four groups: (A) Normal control, (B) TAA (0.03% w/v in drinking water), (C) VN100 (VN 100 mg/kg + TAA) and (D) VN300 (VN 300 mg/kg + TAA). Blood urea and serum creatinine levels were measured,supraoxide dismutase (SOD), catalase (CAT) and malondialdehyde (MDA) levels of renal tissue were assayed. Histopathological analysis together with the oxidative stress nicotinamide adenine dinucleotide phosphate (NADPH) oxidase p22phox in kidney sections were examined in all experimental groups.

**Results:**

Blood urea and serum creatinine levels were increased in TAA group as a result of the nephrotoxicity compared to the VN100 and VN300 groups where, the levels were significantly decreased (*p* < 0.05). Renal MDA level was significantly decreased (*p* < 0.05) in the VN-treated groups with increased CAT and SOD activities compared to the TAA group. Light microscopic examination of renal tissues stained by H&E stain and Masson’s Trichrome for TAA-treated groups revealed severe histopathological changes, whereas specimens obtained from VN-treated groups showed only mild changes. These findings were supported by immunohistochemical results.

**Conclusions:**

VN extract acts as a natural potent antioxidant to prevent ongoing TAA-induced nephrotoxicity in rats, both biochemically and morphologically.

## Background

Kidneys are highly vulnerable to damage caused by reactive oxygen species (ROSs), likely due to oxidative stress by polyunsaturated fatty acids in the composition of renal lipids [1]. This damage can also be caused by a high volume of blood flowing through it, and filtering large amounts of toxins, which can concentrate in kidney lobules [2]. The kidney’s response to toxicants varies by multiple morphological patterns beginning with tubular or interstitial changes to nephropathy [3]. It has been strongly implicated that (NADPH) oxidase, as a major source of ROSs production in the kidney [4] could have a role the in development of renal oxidative damage.

Nephrotoxicity is a poisonous effect due to drugs and its overdose on the kidneys. Thioacetamide (TAA) is an organic compound with the formula CH_3_CSNH_2_. It is originally used as a fungicide, and is a potent hepatotoxin [5]. It can serve as a source of sulphur in the synthesis of organic compounds such as rubber chemicals, curing agents, cross linking agents, metallurgy, pesticides, and pharmaceuticals [5]. TAA is the most potent nephrotoxic substance because of its rapid elimination and cumulative injury when it is given intermittently,presumably by free radical-mediated lipid [2,6]. Metabolic studiesof TAA-induced tissue damage suggest that TAA is metabolized by the mixed function oxidase system to its toxic metabolites sulfine (sulfoxide) and sulfene (sulfone)which are then distributed among several organs,including plasma, liver, kidney, bone marrow, adrenals and other tissues [7]. Later, TAA undergoes an extensive metabolism to acetate and it is excreted through the urine within 24 hours [5,8].

*Vitex negundo* (VN) is commonly called five leaved chaste tree. It is a large aromatic shrub or small and slender with quadrangular branchlets, about 2 to 5 m in height, distributed mainly in tropical to temperate regions, especially in Malaysia, India at the warmest zones and Western Himalayas [9].

The leaves have a typical five foliolate pattern in palmate arrangement measuring4-10 cm long and bluish purple in colour which become dark when ripened. The whole parts of the plant have shown to be a potent source of natural antioxidants [10], 1, 2 di-substituted idopyranose, the isolated compound from VN extract has shown protection of hepatocytes, nephrocytes and pancreatic β-cells in streptozotocin-induced diabetes, probably by its action against NF-kB and induced-nitric oxide synthase iNOS mediated inflammation [11]. A preliminary acute toxicity study of ethanolic leaf extract of VN in albino rats by oral rout carried out by Tandon, 2005 [12] found it to be practically nontoxic, as its LD50 dose was recorded as 7.58 g/Kg body weight with no histomorphological changes in liver, kidney, stomach, heart and lung at any dose of the extract studied.VN has various traditional uses in treating stomach-ache, eye disease, inflammation, enlargement of spleen, bronchitis, asthma and painful teething in children. It is also used as an antihelmintic, promoting hair growth [13], and the juice of the leaves used in treating ulcers and swelling of joints [14]. Literature review reveals that the plant of VN possesses analgesic and antinociceptive activity [15], hepatoprotective activity against TAA [16], antituberculardrugs [12], CCl_4_[17] and Ibuprofen via inhibition of lipid peroxidation [18].

In this study, VN extract was utilized in a rat model to evaluate its possible protective effects on TAA-induced nephrotoxicity. Data were collected on serum creatinine level, blood urea level, kidney and body weight ratio, glutathione content, and lipid peroxidation in the kidney tissues, as well as determination of p22phox expression and histopathological changes in the kidneys after administering VN extract in the adult male SD rats.

## Methods

### Collection and preparation of plant extract

Fresh leaves of VN plant were obtained from Kampung Baru, Sungai Ara, Penang, Malaysia. The botanical identity was determined and authenticated in the Department of Pharmacy, Faculty of Medicine, University Malaya, Kuala Lumpur, Malaysia with voucher specimen number (KLU 34968). The plant was dried and grounded to a fine powder. Next, the powder was homogenized in 95% ethanol at a ratio of 1:10 of plant to ethanol, and left to soak for four days at 25°C with occasional shaking and stirring. Later, the mixture was filtered through filter paper, and the resulting liquid was concentrated at a reduced pressure at 45°C to obtain a dark gummy–green extract. The percentage yield of VN crude extract was 18%. The extract was then dissolved in Tween 20 (10% w/v) and administered orally to rats in concentrations of 100 and 300 mg/kg body weight.

### Preparation of TAA

TAA (from Sigma-Aldrich, Switzerland) and all other chemicals used were of analytical grade and purchased mostly from Sigma-Aldrich and Fisher. TAA stock solution was prepared by dissolving30 mg pure TAA which is in crystal form in 100 ml distilled water (0.03% w/v) until all the crystals were dissolved. The solution was given to the rats as their daily drinking water [19].

### Experimental animals

A healthy adult male *Sprague Dawley* rats weighing 180–200 gm were obtained from the Animal House Unit, Faculty of Medicine, University of Malaya, Malaysia. They were kept in wire-bottomed cages at 25 *±* 3°C, at 50–60% humidity, and a 12 h light-dark cycle for at least a week before the experiment. They were maintained under standard housing conditions with free access to a standard diet and water ad libitum during the experiment. The experimental protocol was approved by the Institutional Animal Care and Use Committee, University of Malaya (UM IACUC) with an ethical no. ANA/18/05/2012/FAAK. Throughout the experiment, all criteria for taking care of animals prepared by the National Academy of Sciences and outlined in the “Guide for the Care and Use of laboratory Animals” were compiled [20].

The animals were randomly divided into four experimental groups, with each group consisting of six rats, and given the following treatments: Group A: Normal control group, received per oral treatment of 10% Tween 20 (5 ml/kg) daily for 12 weeks; Group B: TAA group, received TAA 0.03% w/v in drinking water daily for 12 weeks; Group C: VN100 group, received TAA 0.03% w/v in drinking water and 100 mg/kg body weight of VN extract daily for 12 weeks; Group D: VN300 group,received TAA 0.03% w/v in drinking water and 300 mg/kg body weight of VN extract daily for 12 weeks.

The body weights of the animals were recorded weekly throughout the experimental period starting from Day 0. At the end of the 12^th^ week, the rats were sacrificed 24 hours after the last treatment followed by overnight fasting. The rats were anaesthetized by intramuscular injection of 50 mg/kg ketamine mixed with xylazine 5 mg/kg. Blood samples were collected and serum was obtained for estimation of creatinine and blood urea levels. Kidney samples were dissected, trimmed of connective tissues, washed using normal saline to eliminate blood contamination, dried by blotting with filter paper and weighed. The kidneys were excised into two halves. One-half was kept in isotonic formalin for histopathological assessment and the other half was kept in the freezer under -80°C for preparing kidney homogenate for the malondialdehyde (MDA), catalase (CAT) and supraoxide dismutase(SOD) assays.

### Assessment of renal function

A blood sample was withdrawn through the jugular vein and collected into a plain tube with activated gel for detection of urea and creatinine levels. The samples were allowed to clot, centrifuged and the serum samples were sent for analysis using a standard automated technique in the Central Diagnostic Laboratory (CDL), University of Malaya Medical Centre, according to the procedures described by the manufacturers.

### Preparation of kidney homogenates

Kidney homogenates (10% w/v) were prepared by homogenizing kidney tissue in cold 50 mM potassium phosphate buffer saline (pH 7.4) using a tissue homogenizer (DAIHAN Sci., Seoul, Korea). The cell debris was removed by centrifugation at 4500 rpm for 15 minutes at 4°C using a refrigerated centrifuge Rotofix 32 (Hettich Zentrifugen, Germany). The supernatant was used for estimating the following *in vivo* antioxidant using commercially available kits (Cayman Chemical Company, USA): (MDA) or thiobarbituric acid reactive substance (TBARS) (Item No. 10009055), (CAT) (Item No. 707002) and (SOD) (Item No. 706002). All assays were performed according to the instruction manual of the manufacturers.

### Histopathological assessment

The lower half of the right kidney was examined for histopathological changes. All kidneys specimens were examined under a light microscope. The kidneys were fixed in 10% formalin and then embedded in paraffin wax before sectioning at 5 μm thickness and stained with haematoxylin-eosin and Masson’s trichrome stains. Kidney sections from the six rats in each group were examined by two dependent observers. Morphological analysis there were carried out according to Houghton et al., [21] and were graded as follows: Grade 0 (normal), Grade 1 (when changes were limited to the tubulointerstitial areas of focal granulovascular epithelial cell degeneration and granular debris in the tubular lumina with or without evidence of desquamation in small foci (<1% of total tubule population involved by desquamation), Grade 2 (when tubular epithelial necrosis and desquamation were easily seen but involved less than half of the cortical tubules), Grade 3 (when more than half of the proximal tubules showing necrosis and desquamation, but with intact tubules easily identified, and Grade 4 (when there was complete or almost complete proximal tubular necrosis).

### Immunohistochemical study

For the quantitative determination of anti-p22 phox antibody, immunohistochemistry kit for p22 phox (CS9): sc-130551 (Santa Cruz Biotechnology, INC.) was used. Briefly, sections were deparaffinized in xylene and hydrated in a series of graded alcohol. Deparaffinized sections were treated in a microwave oven in a citrate buffer at 95°C for 15 minutes, and immersed in 3% hydrogen peroxide in methanol for 10 min to abolish endogeneous peroxidase activities. The sections were immersed in normal goat serum for 15 min, incubated withmouse monoclonal antibody as a marker for NADPH oxidase subunits (diluted to 1:50) at 37°C for 60 min, and then incubated with goat anti-mouse IgG conjugated with horseradish\ peroxidise (HRP) (diluted to 1:200). The reaction products were visualized using 3-30-deiaminobenizidine tetrahydrochloride and hydrogen peroxide. A cytoplasmic brown granule was marked as positive expression of p22 phox.

### Statistical analysis

All data were expressed as mean ± standard error of the mean (SEM) and statistical analysis was performed using SPSS for Windows version 17.0 (SPSS Inc. Chicago, IL, USA). One-way analysis of variance (ANOVA) followed by Bonferroni post hoc test was applied to test for statistically significant differences between groups at *p* < 0.05.

## Results

### Gross pathology

During the course of this study, continuous daily administration of 0.03% w/v TAA in drinking water was not associated with animal mortality. The external appearance of the kidneys from animals treated with TAA in drinking water as well as VN-treated groups revealed a smooth, glistening capsule with no petechial hemorrhage was noted.

### Body and kidney weight

In the present study, the body weight of rats administered with TAA were reduced significantly (*p <* 0*.*05) in comparison to the normal control group. Although there was increased in body weight of VN300 + TAA and VN100 + TAA groups, the increased in body weight was not significant (*p <* 0*.*05) compared to TAA group. Concurrently, no noticeable concurrent significant difference in the weights of the kidneys for all groups was shown (Table 1).

**Table 1:** Effect of VN on body and kidney weights

**Group**	**Body weight (g)**	**Kidney weight (g)**
**Normal control**	219.8 ± 29.88^**^	0.95 ± 0.23
**TAA**	177.5 ± 7.1^*^	0.93 ± 0.119
**VN100 + TAA**	199.3 ± 15.98	0.94 ± 0.22
**VN300 + TAA**	204.3 ± 10.70	0.99 ± 0.29

Data were stated as mean *±* SEM. Means with different superscripts are significantly different. ^*^P < 0*.*05 versus normal control group and ^**^P < 0*.*05 versus TAA group.

### Biochemical determination

Rats from the TAA-treated group exhibited significantly increased (*p <* 0*.*05) levels of blood urea and serum creatinine compared to the normal control group. On the other hand increases in these parameters were prevented by concurrent treatment of animals with VN 100 mg/ kg, and more effectively, with VN 300 mg/kg which resulted in nearly normalized levels of these parameters (Table 2).

**Table 2:** Effect of VN on blood urea and serum creatininelevels

**Group**	**Urea (mmol/L)**	**Creatinine (mol/L)**
**Normal control**	4.46 ± 0.45^**^	23 ± 1.7^**^
**TAA**	8.73 ± 1.31^*^	52.3 ± 7.5^*^
**VN100 + TAA**	6.28 ± 0.59^*,**^	35.66 ± 7.3^*,**^
**VN300 + TAA**	5.73 ± 0.973^**^	29.33 ± 3.4^**^

Data were stated as mean *±* SEM. Means with different superscripts are significantly different. ^*^P < 0*.*05 versus normal control group and ^**^P < 0*.*05 versus TAA control group.

CAT and SOD are some of the enzymes of the intrinsic antioxidant defense system [22] which are responsible for the dissemination of free radicals such as superoxide radicals. Table 3 shows that SOD was significantly (*p* < 0.05) increased in high and low dose VN-treated groups but decreased in the TAA group due to long term excretion of free radicals. Generally, rats treated only with TAA, had significantly higher levels of MDA (*p* < 0.05) than normal rats and the other experimental treated groups. Notably, experimental rats treated with low dose and high dose VN extract had significantly lower levels (*p* < 0.05) of renal MDA compared to TAA group. These results suggest that treatment with VN extract may protect renal tissue from further damage.

**Table 3:** **Effects of VN on some *in vivo *** **antioxidant parameters in all experimental groups**

**Group**	**CAT**	**SOD**	**MDA**
	**(nmol/mg protein)**	**(U/mg protein )**	**(nmol/mg protien)**
**Normal control**	27.24 ± 34.32	32.23 ± 7.14	38.74 ± 2.61
**TAA**	21.57 ± 24.87^*^	22.3 ± 8.58^*^	107.14 ± 3.71^*^
**VN100 + TAA**	24.75 ± 105.09	26.73 ± 0.93^**^	78.85 ± 2.26^**^
**VN300 + TAA**	25.26 ± 56.01	27.47 ± 3.44^**^	54.35 ± 1.73^**^

Data were stated as mean *±* SEM. Means with different superscripts are significantlydifferent. ^*^P < 0*.*05 versus normal control group and ^**^P < 0*.*05 versus TAA control group.

### Histopathological findings

**Figure 1:**
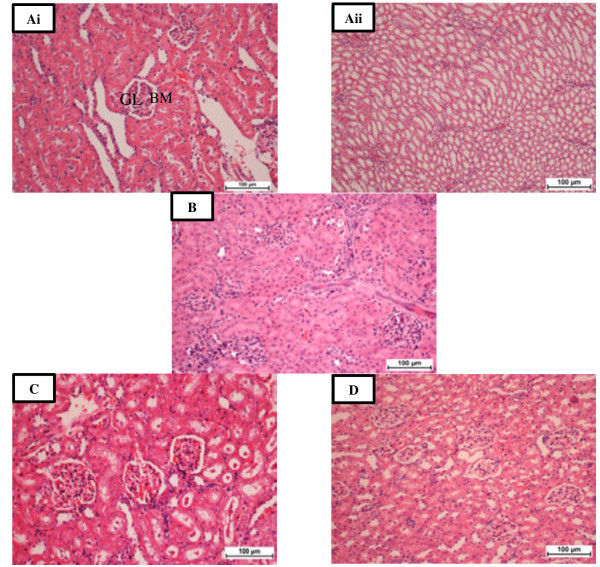
**Photomicrographs of renal sections in all experimental groups. Ai**: Normal control-cortical area, showing normal glomeruli (GL) with intact Bowman’s capsule (BM) and proximal convoluted tubules. **Aii**: Normal control-Medullary area, showing intact collecting ducts. **B**: TAA group-Cortical area, showing swollen and necrotic tubular cells, inflammatory cells infiltrate the GL and intertubular area and thinning-out of the BM. **C**: VN 100 group- Cortical area exhibiting mild tubular epithelial changes with intratubular casts and mild intertubular inflammatory cells infiltration and GL look normal. **D**: VN 300 group - Cortical area, showing both GL and renal tubules appearing normal. H & E stain. Bar = 100 μm.

**Figure 2:**
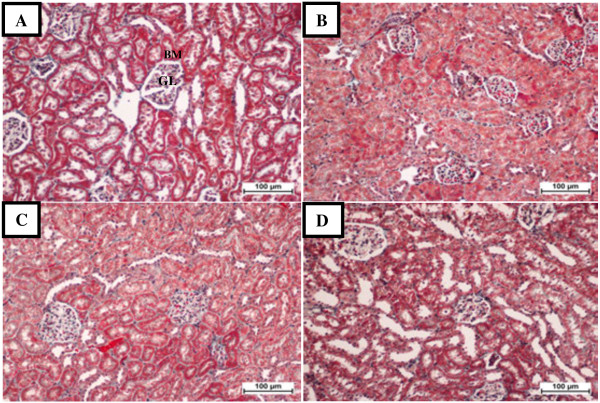
**Photomicrographs of renal sections stained with Masson’s trichrome of the different groups. A**: Normal control, showing normal glomeruli (GL) in the cortical area, with intact Bowman’s capsule (BM) and proximal convoluted tubules. **B**: TAA group, showing renal cortical tissue with swollen and necrotic tubular cells, **C**: VN100 group, showing mild tubular epithelial changes in renal cortical tissue. **D**: VN300 group, showing renal cortical tissue with both GL and tubules appearing normal. Bar = 100 μm.

Histopathological examination of sections from rat kidneys treated with TAA showed impaired renal morphology throughout with severe and generalized tubular epithelial cell necrosis associated with diffuse tubular swelling, glomerular congestion and inflammatory cell infiltration. Kidneys from animals concurrently treated with VN 100 mg/kg body weight + TAA 0.03% in their drinking water showed mild to moderate inflammatory cell infiltration and tubular epithelial cell necrosis (Figure 1C), while concurrent treatment with VN 300 mg/kg body weight provided the best morphological protection against TAA-induced renal damage (Figure 1D and Figure 2D).

## Discussion

Various useful drugs like acetaminophen, gentamicin and some environmental and industrial toxicants can cause severe renal damage through activation of these drugs to highly reactive free radicals [23]. One of the most extensively studied chemicals and industrial toxicants is TAA. TAA is known to induce centrilobular hepatic necrosis, liver cirrhosis, hepatocellular carcinoma and bile duct proliferation [5,8,16,24,25] with injury to the terminal portion of the proximal renal tubule [7]. After administration of TAA, this chemical undergoes an extensive metabolism forming sulfine (sulfoxide) and sulfene (sulfone). Both are circulated through several vital organs in body before finally being transformed into acetate and excreted into urine within 24 hours [8].

TAA administration (0.03% w/v) in drinking water for 12 weeks found to cause renal toxicity as assessed by biochemical analysis of urea and creatinine, MDA and antioxidant status of SOD and CAT. As a measure of renal function status, blood urea and creatinine are often regarded as reliable markers [26]. The increased urea and creatinine levels resulting from TAA administration in this study (Table 2), are in agreement with a previous study done by Begum et al., [2]. High values of blood urea and serum creatinine indicate renal damage [27,28] and this may be correlated with the significant and progressive weight loss in the TAA group (Table 1). As for the VN100 and VN300 groups, although there was increased body weight in both groups, the increase was not significantly different compared in the TAA group (Table 1).

From this study, pretreatment with VN 100 mg/kg extract could partly prevent TAA-induced kidney damage as shown by the decreasing levels of urea and creatinine. These parameters were almost significantly normalized by the administration of VN 300 mg/kg extract (Table 2). This result is consistent with many previous studies done using other traditional plants [29], and is strongly attributed to the scavenging free radicals and reduced lipid peroxidation mechanisms.

Free radicals are believed to play a major role in the development of TAA-induced toxicity [30], hence, when oxidative stress is overwhelming, the various inherent defense mechanisms such as the antioxidant defense mechanisms, intercellular concentration of CAT and SOD activities become significantly impaired and insufficient [31]. This is because during oxidative stress, the body uses its defense mechanism to minimize the process of lipid peroxidation using these antioxidant enzymes, thus, the activity of these enzymes becomes higher in early stages of TAA induction. When the oxidative insult was continued for a long period, the enzymes become depleted and unable to fight against the free radicals, suggesting advance stages of TAA-induced peroxidation. This was evident in the present study with the significant increase (*p <* 0*.*05) in MDA level, renal damage in TAA group compared to the normal control group.

Several phytochemical studies have revealed the presence of volatile oils, lignans, flavonoid like flavones, luteolin-7-glucoside and glycosides in VN [9]. In addition, VN ethanolic extract possesses radical scavenging activity probably due to higher concentration of flavonoids and alkaloids [32]. This was evident in the increased (CAT and SOD) levels in this study as well as the decreased in MDA level in the VN*-*treated groupsas shown in Table 3. Presumably, this is due to the antioxidant properties of the plant extract, which may account for the nephroprotective action of VN against TAA-induced nephrotoxicity experimental models.

**Figure 3:**
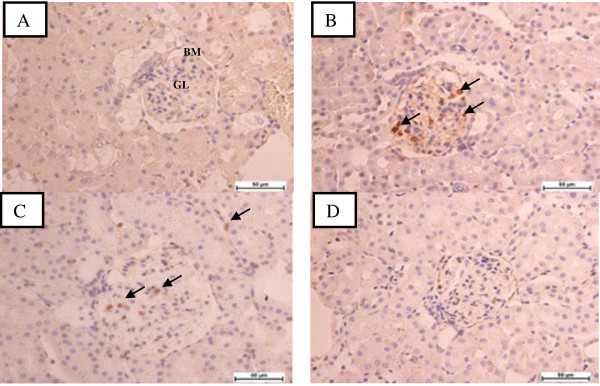
**Photomicrographs of renal sections for immunohistochemical evaluation of 22phox protein expression in all experimental groups. A**: Normal control, showing normal glomerulus (GL) in the cortical area, with intact Bowman’s capsule (BM) and normal tubulointerstitium. **B**: TAA group showed p22phox was markedly expressed in glomerulus and interstitium, **C**: VN100 and **D**: VN300 groups, showed marked reduction in p22phox protein expression. Bar = 50 μm.

TAA is toxic to selected populations of cells (hepatocytes, proximal convoluted tubular cells in kidney and cortical thymocytes) [7], and several animals studies have shown that toxin-induced renal tubular damage plays a crucial role in the reduction of glomerular filtration rate either through obstruction and back leak of renal filtrate or secondary to ROS [33]. The p22phox is one of the subunits of the NADPH oxidase which generates ROS that plays an important role in the development of many kidney diseases [4,34]. Thus, VN is important in protecting the kidney from TAA-induced injury, through the improvement in renal function and antioxidant status. Furthermore, these findings were corroborated by the renal histological and immunohistochemical results, which revealed more extensive and marked tubular and glomerular damage with apparent expression of p22phox protein in the TAA-treated group. Similar changes were also reported by Edward et al., [7] demonstrating structural changes in renal tissue. However, in the VN-treated groups, renal microscopic changes were alleviated and the renal histological architecture was almost normalised, especially, at the maximum dose of 300 mg/kg as shown in Figures 1, 2, 3 and Table 4. Although the exact mechanism of TAA-induced nephrotoxicity is not well understood, but based on several previous studies [27,28,35,36] we can postulate that the protective effect of VN extract is via its antioxidant and/or free radical scavenging activities due to its high concentration of flavonoids and alkaloids content [9].

**Table 4:** Histopathological grading of the kidney sections for all experimental groups

**Pathological grading**							
**Group**	**N**	**0**	**I**	**II**	**III**	**IV**	**Average of grades**
**Normal**	6	6	0	0	0	0	0.0^**^
**TAA**	6	0	0	0	2	4	3.33 ± 0.51^*^
**VN 100 + TAA**	6	0	1	1	4	0	2.00 ± 0.63^**^
**VN 300 + TAA**	6	1	3	2	0	0	1.16 ± 1.34^**^

Data were stated as mean *±* SEM. Means with different superscripts are significantly different. ^*^P < 0*.*05 versus normal control group and ^**^p < 0*.*05 versus TAA control group.

## Conclusions

The overall results from this study demonstrated TAA-induced renal injury by biochemical analysis, histopathological features and immunohistochemistry analysis. The concurrent treatment with VN extract clearly provided a considerable degree of protection in a dose-dependent manner against the deleterious renal side effects of TAA. In conclusion, VN could act on the kidney as a potent natural antioxidant to prevent ongoing TAA*-*induced nephrotoxicity, thus, these results constitute a lead towards discovering a novel herb in traditional and complementary medicine.

## Competing interests

The authors declare that they have no competing interest.

## Authors’ contributions

Conceived and designed the experiments: FK, NMK, MAA and WAY. Performed the experiments FAK, WAY. Analyzed the data: FK and WAY. Contributed reagents, materials and analysis tools: FK, NMK, MAA and WAY. Wrote the paper: FK and WAY. Editing the paper: FK, NMK, MAA and WAY. All authors read and approved the final manuscript.

## Pre-publication history

The pre-publication history for this paper can be accessed here:

http://www.biomedcentral.com/1472-6882/13/294/prepub
